# Click It or Ticket Campaign — May 19–June 1, 2014

**Published:** 2014-05-16

In 2012, approximately 21,000 passenger vehicle occupants died in motor vehicle crashes in the United States; 52% were unrestrained at the time of the crash (1). An additional estimated 2.6 million nonfatal injuries from motor vehicle crashes were treated in emergency departments (2). Seat belt use in the United States reached 86% in 2012, but millions of U.S. residents continue to travel unrestrained (3). Using a seat belt is one of the most effective ways to prevent serious injury or death in the event of a crash. Seat belts saved an estimated 12,174 lives in 2012. If everyone had been buckled up, an estimated 3,031 additional lives could have been saved (1).

Click It or Ticket, a national campaign coordinated annually by the National Highway Traffic Safety Administration (NHTSA) to increase the proper use of seat belts, takes place May 19–June 1, 2014. Law enforcement agencies across the nation will conduct intensive, high-visibility enforcement of seat belt laws during daytime and nighttime hours. Nighttime enforcement of seat belt laws is encouraged because seat belt use is lower at night (4). Campaign activities in 2014 will focus primarily on men aged 18–34 years, who research has shown are less likely to wear seat belts (1). Additional information about 2014 Click It or Ticket campaign activities is available from NHTSA at http://www.nhtsa.gov/Driving+Safety/Occupant+Protection. Additional information on preventing motor vehicle crash related injuries is available from CDC at http://www.cdc.gov/motorvehiclesafety.
